# Superior virologic and treatment outcomes when viral load is measured at 3 months compared to 6 months on antiretroviral therapy

**DOI:** 10.7448/IAS.18.1.20092

**Published:** 2015-09-23

**Authors:** Bernhard Kerschberger, Andrew M Boulle, Katharina Kranzer, Katherine Hilderbrand, Michael Schomaker, David Coetzee, Eric Goemaere, Gilles Van Cutsem

**Affiliations:** 1 Médecins sans Frontières, Khayelitsha, Cape Town, South Africa; 2 Centre for Infectious Disease Epidemiology and Research, School of Public Health and Family Medicine, Faculty of Health Sciences, University of Cape Town, Cape Town, South Africa; 3 Department of Infectious Disease Epidemiology, London School of Hygiene and Tropical Medicine, London, UK; 4 South African Medical Unit, Médecins sans Frontières, Cape Town, South Africa

**Keywords:** viral load, HIV, treatment switching, virologic failure

## Abstract

**Introduction:**

Routine viral load (VL) monitoring is utilized to assess antiretroviral therapy (ART) adherence and virologic failure, and it is currently scaled-up in many resource-constrained settings. The first routine VL is recommended as late as six months after ART initiation for early detection of sub-optimal adherence. We aimed to assess the optimal timing of first VL measurement after initiation of ART.

**Methods:**

This was a retrospective, cohort analysis of routine monitoring data of adults enrolled at three primary care clinics in Khayelitsha, Cape Town, between January 2002 and March 2009. Primary outcomes were virologic failure and switch to second-line ART comparing patients in whom first VL done was at three months (VL3M) and six months (VL6M) after ART initiation. Adjusted hazard ratios (aHR) were estimated using Cox proportional hazard models.

**Results:**

In total, 6264 patients were included for the time to virologic failure and 6269 for the time to switch to second-line ART analysis. Patients in the VL3M group had a 22% risk reduction of virologic failure (aHR 0.78, 95% CI 0.64–0.95; *p*=0.016) and a 27% risk reduction of switch to second-line ART (aHR 0.73, 95% CI 0.58–0.92; *p*=0.008) when compared to patients in the VL6M group. For each additional month of delay of the first VL measurement (up to nine months), the risk of virologic failure increased by 9% (aHR 1.09, 95% CI 1.02–1.15; *p*=0.008) and switch to second-line ART by 13% (aHR 1.13, 95% CI 1.05–1.21; *p*<0.001).

**Conclusions:**

A first VL at three months rather than six months with targeted adherence interventions for patients with high VL may improve long-term virologic suppression and reduce switches to costly second-line ART. ART programmes should consider the first VL measurement at three months after ART initiation.

## Introduction

An estimated 32.6 million people live with HIV/AIDS worldwide and 11.7 million received antiretroviral treatment (ART) in middle- and low-income countries in 2013 [1]. Antiretroviral treatment is effective [2] and feasible in resource-constrained settings, and large HIV programmes report good patient retention [3–5]. In 2012, more than 2 million people living with HIV/AIDS (PLWHA) in South Africa received ART and scaling up of HIV services continues [6].

Major challenges, however, exist in the preservation of potent first- and second-line treatment regimens in resource-constrained settings, where third-line treatment options remain expensive and difficult to access. Maintaining and monitoring proper adherence to therapy is an increasingly important priority for ART programmes. Sub-optimal adherence at the individual level can lead to treatment failure, accumulation of drug resistance mutations [7], and may result in excess morbidity and mortality.

Routine viral load (VL) monitoring during treatment is regarded as the gold standard to assess treatment adherence and virologic failure [8,9]. VL monitoring has been shown to be feasible and sustainable in some resource-constrained settings [3–5]; however, the optimal timing and frequency of VL monitoring has yet to be determined.

Early adherence to therapy predicts short- and long-term virologic suppression [10,11] and early detection of sub-optimal adherence accompanied by targeted adherence interventions may lead to better virologic outcomes [12,13]. National and international treatment guidelines generally recommend the first VL as late as six months after ART initiation [8,14]. We hypothesized that a VL at three months (VL3M) after ART initiation would improve virologic outcomes by preventing the emergence of resistance through earlier adherence support interventions, when compared to a VL at six months (VL6M).

## Methods

### Study setting

This study was done in Khayelitsha township, situated in the outskirts of Cape Town, South Africa. The estimated antenatal HIV seroprevalence was 26% in 2010 and the TB annual incidence is over 1500/100,000 [15]. Médecins Sans Frontières and the Provincial Government of the Western Cape have provided ART in three community health centres in Khayelitsha since 2001. In 2011, 20,000 were still in care on ART [15], with 4.5% on a second-line treatment regimen (routine program data, Khayelitsha, 2012).

Between 2002 and 2009, patients with CD4 counts ≤200 cells/µL or WHO stage 4 were initiated on ART, most commonly lamivudine (3TC) with zidovudine (AZT) or stavudine (d4T) combined with nevirapine (NVP) or efavirenz (EFV). Routine VL (EasyQ HIV-1 assay; bioMerieux, Boxtel, The Netherlands) and CD4 cell count (single platform panleucogating method) monitoring was performed at six-month intervals. Until 2005/2006, VL was measured first at three months after ART initiation to assess early treatment adherence; after this period the first VL was performed at six months after ART initiation as per new national treatment guidelines. Patients with an elevated VL (≥400 copies/mL) underwent enhanced adherence counselling: HIV counsellors reassessed the patient's knowledge, attitude and beliefs towards treatment, and addressed obstacles towards optimal drug adherence. A follow-up VL was performed three months after the first elevated VL; if the follow-up VL was ≥5000 copies/mL the definition of virologic failure was met (i.e. two consecutive elevated VL measurements: first VL≥400 copies/mL and second VL≥5000 copies/mL). When the second VL was between 400 and 5000 copies/mL adherence counselling and intensified follow-up continued. Patients with confirmed virologic failure were switched to a second-line ART regimen, consisting of lopinavir/ritonavir with two or more nucleoside reverse transcriptase inhibitors. All services, including drugs and laboratory, were provided free of charge. Data were recorded by clinicians on structured clinical records, then prospectively entered by data capturers on site into a regularly validated database used for both monitoring and research.

### Key variables and definitions

Patients aged 16–60 years who initiated first-line triple ART regimen between 1 January 2002 and 31 March 2009 were eligible for analysis. The first VL measurement at either three or six months after ART initiation was performed in one of the three study clinics. VL3M was defined as any measurement recorded between 2.5 and 4.5 months, and VL6M between 4.5 and 9 months after ART initiation. If two or more VLs were recorded in the same time period, then the time of the first measurement was considered.

Baseline demographic and clinical data at the time of ART initiation included gender, age, combined WHO clinical stages 1/2 or 3/4, study clinic, baseline NNRTI drug used and date of ART initiation. Baseline absolute CD4 counts were those taken between 12 months prior or one week after ART initiation; in case of two or more recorded results, the result closest to ART initiation date was used.

The primary outcomes were virologic failure, defined as occurrence of the second consecutive detectable VL (first VL≥400 and second consecutive VL≥5000), and switch to second-line ART, defined as any change from a first-line to a second-line ART regimen containing the drugs lopinavir/ritonavir and two or more nucleoside reverse transcriptase inhibitors.

Follow-up time started at nine months after ART initiation. Patients who were lost to follow-up (LTFU), transferred-out or died within nine months of ART initiation were excluded from the analysis. LTFU was defined as no clinical visits to any of the clinics for more than three months. Patients without documentation of a primary outcome who remained in care were censored at the end of the observation period (31 December 2009). Patients on second-line treatment without recorded virologic failure were censored at the date of switch to second-line ART for the virologic failure analysis. Patients with another outcome (death, transfer out, LTFU) occurring before virologic failure and treatment switching were considered at risk and right censored at date of the event. In addition, the analysis of time to virologic failure was corrected for treatment interrupters and VL re-suppressors. First, elevated VLs after treatment interruptions (defined as not attending the clinic for more than 2.5 months but having returned into care before the end of the study) were not counted and only patients with two consecutive detectable VLs after return to care were recorded as having virologic failure. Second, patients not on second-line ART but with a re-suppressed VL after recorded virologic failure were counted as virologic suppressors until next virologic failure occurred.

### Statistical analysis

Data were checked for duplications, inconsistencies and errors. Baseline characteristics of the VL3M and VL6M groups were compared using Wilcoxon rank-sum for continuous and Chi squared tests for categorical variables. Kaplan–Meier estimates were calculated for time to virologic failure and switch to second-line ART. Potential confounders were determined *a priori* through directed acyclic graphs (DAGs): sex, age, WHO stage, CD4 count, clinic, baseline NNRTI and calendar time of ART initiation. Multivariate Cox proportional hazard models were fitted to determine the association between timing of first VL and the outcomes. Baseline CD4 count (per 50 cells/µL change), age (per 10 years change) and calendar time of ART initiation (per 90 days change) were included as continuous variables. Variance inflation factors (VIF) were calculated to assess collinearity of independent predictors. Variables were tested for interactions. The proportional-hazards assumption (PHA) was tested globally based on Schoenfeld residuals, and variables were categorized in case of violation. In a supplementary analysis, the timing of first VL measurement was included as a continuous time variable, indicating the number of days since ART initiation. All data were analyzed using STATA version 11.0 (Stata-Corp Inc., College Station, TX, USA).

### Sensitivity analyses

Categorization is generally not recommended as it may decrease power and efficiency, and may also introduce additional bias [16–18], yet categorization is widely used in the medical literature [19]. Therefore, we did a first sensitivity analysis in which continuous variables were converted into multiple categories based on cut-points used in other studies [20,21]. As a second sensitivity analysis, we excluded the nine months transition period from old to new guidelines (1 July 2005–31 March 2006) and only VLs done according to guideline were considered. Then, study follow-up time was restricted to two years to ensure that the prolonged follow-up time in VL3M group does not influence the estimates. Finally, the CD4 variable was categorized and an extra category was created for missing values.

### Ethics

All data were anonymised prior to analysis. Ethical approval was obtained for use of routine cohort data from the University of Cape Town Research Ethics Committee.

## Results

### Baseline characteristics

In total, 6841 patients were eligible for the time to virologic failure analysis and 6848 for the switch to second-line ART analysis. Baseline characteristics for the switch to second-line ART analysis are presented in Table 1. In total, 2589 (37.8%) patients were in VL3M group. There were no significant differences in gender, clinic attended or baseline NNRTI regimen. Patients in the VL3M group had more advanced immune-suppression, with lower CD4 counts at initiation of ART (median: 99.5 vs. 134 cells/µL, *p*<0.001) and more advanced WHO stage (85.5% vs. 71.5% in combined stages 3 and 4, *p*<0.001). Patients were more likely to get the first VL measurement at three months in the earlier calendar years of 2002 to 2005, consistent with policy at different time points (*p*<0.001).

**Table 1:** Baseline characteristics of patients with the first VL measurement at either 3 or 6 months after ART initiation in three community health centres in Khayelitsha, Cape Town, South Africa

	All	VL 3 months	VL 6 months	*p*
Total enrolled; *n*	6848	2589 (37.8)	4259 (62.2)	
Median follow-up time; years (IQR)	2.2 (0.9–3.4)	3.6 (1.9–4.5)	1.7 (0.7–2.6)	
Sex; *n* (%)				0.470
Female	4816 (70.3)	1834 (70.8)	2982 (70.0)	
Male	2032 (29.7)	755 (29.2)	1277 (30.0)	
Age; years, Median (IQR)	32 (28–38)	32 (28–38)	33 (28–39)	<0.001
CD4 count; (cells/µL), Median (IQR)	119 (56–175)	99.5 (44–159)	134 (66–183)	<0.001
WHO stage; *n* (%)				<0.001
I+II	1590 (23.2)	375 (14.5)	1215 (28.5)	
III+IV	5257 (76.8)	2214 (85.5)	3043 (71.5)	
Clinic; *n* (%)				0.409
I	2514 (36.7)	930 (35.9)	1584 (37.2)	
II	1540 (22.5)	577 (22.3)	963 (22.6)	
III	2794 (40.8)	1082 (41.8)	1712 (40.2)	
Baseline NNRTI; *n* (%)				0.654
Nevirapine	3510 (51.3)	1336 (51.6)	2174 (51.0)	
Efavirenz	3338 (48.7)	1253 (48.4)	2085 (49.0)	
ART initiation; year, *n* (%)				<0.001
2002	170 (2.5)	143 (5.5)	27 (0.6)	
2003	306 (4.5)	271 (10.5)	35 (0.8)	
2004	875 (12.8)	752 (29.1)	123 (2.9)	
2005	1391 (20.3)	1058 (40.9)	333 (7.8)	
2006	1639 (23.9)	148 (5.7)	1491 (35.0)	
2007	974 (14.2)	77 (3.0)	897 (21.1)	
2008	1168 (17.1)	102 (3.9)	1066 (25.0)	
2009	325 (4.8)	38 (1.5)	287 (6.7)	

Data for treatment switching analysis are presented; baseline characteristics for virologic failure analysis are not presented as the patient sample differs by only seven patients.

**Figure 1:**
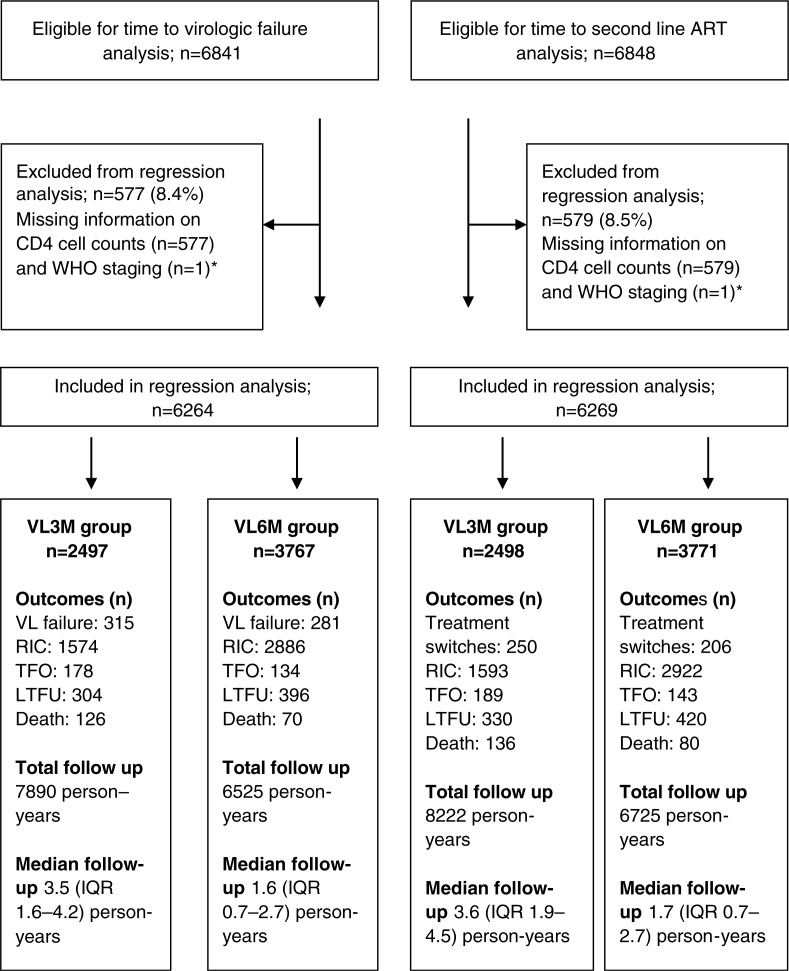
Flow chart of patients eligible for baseline and regression analyses who initiated ART in three community health centres in Khayelitsha, Cape Town, from 1 January 2002 to 31 March 2009. *One patient with missing information on WHO staging also had missing information on CD4.

The median follow-up (starting from nine months after ART initiation) was 2.1 (IQR 0.8–3.4) and 2.2 (IQR 0.9–3.4) years for time to virologic failure and switch to second-line ART, respectively. During the follow-up period, 624 (9.1%) patients had recorded virologic failure and 479 (7.0%) were switched to second-line ART. The crude rates of virologic failure and switch to second-line ART during the study period were 4.1 (95% CI 3.8–4.4) and 3.0 (95% CI 2.8–3.3) per 100 person-years. Due to missing information on baseline CD4, 577 (8.4%) and 579 (8.5%) observations were omitted for time to virologic failure and switch to second-line ART, leaving 6264 and 6269 patients for multivariate analysis. Figure 1 shows a detailed description of patient outcomes and follow-up time for each VL group. The VL3M group included less study participants but median and total follow-up time was longer when compared to VL6M group.

### Outcomes

Patients in the VL3M group were less likely to experience virologic failure (adjusted hazard ratio (aHR) 0.78, 95% CI 0.64–0.95, *p*=0.016) or being switched to second-line ART (aHR 0.73, 95% CI 0.58–0.92, *p*=0.008) (Table 2). For each additional month of delay of the first VL measurement (between 2.5 and 9 months) the risk of virologic failure increased by 9% (aHR 1.09, 95% CI 1.02–1.15; *p*=0.008) and the risk of switch to second-line ART by 13% (aHR 1.13, 95% CI 1.05–1.21; *p*<0.001) (Table 3). Tables of the detailed models are available in Supplementary Files 1 and 2.

**Table 2:** Unadjusted and adjusted hazard ratios of virologic failure (*n*=6264) and treatment switching (*n*=6269)

	Univariate analysis			Multivariate analysis			
		
	cHR	95% CI	*p*	aHR	95% CI	*p*	PHA, VIF
Virologic failure							0.493, 1.01–1.16
6 months	1		0.257	1		0.016	
3 months	1.10	0.93–1.29		0.78	0.64–0.95		
Treatment switching^a^							0.187, 1.01–1.23
6 months	1		0.803	1		0.008	
3 months	1.02	0.85–1.23		0.73	0.58–0.92		

cHR, crude hazard ratio; aHR, adjusted hazard ratio; PHA, *p* value for the global proportional-hazards assumption; VIF, range of variance inflation factors.

a The variable age was categorized because the global proportional-hazards assumption was violated with age as a continuous variable (*p*= 0.039).

**Table 3:** Unadjusted and adjusted hazard ratios of virologic failure (*n*= 6264) and treatment switching (*n*= 6269)

	Univariate analysis			Multivariate analysis			
		
	cHR	95% CI	*P*	aHR	95% CI	*p*	PHA, VIF
Virologic failure							0.531, 1.01–1.20
Timing of VL^a^	0.990	0.986–0.995	<0.001	1.09	1.02–1.15	0.008	
Treatment switching							0.197, 1.01–1.23
Timing of VL^a^^b^	0.992	0.987–0.997	0.003	1.13	1.05–1.21	<0.001	

The timing of first VL done after ART initiation was included as a continuous time variable, indicating the number of months since ART initiation. cHR, crude hazard ratio; aHR, adjusted hazard ratio; PHA, p value for the global proportional-hazards assumption; VIF, range of variance inflation factors.

a HR of virologic failure or treatment switch for per month of delay of the first VL done since ART initiation (between 2.5 and 9 months);

b the variable age was categorized because the proportional-hazards assumption was violated with age as a continuous variable (*p*=0.045).

### Sensitivity analyses

Effect estimates in sensitivity analyses were generally consistent with findings from the main analyses. After categorization, the aHRs were 0.83 (95% CI 0.66–1.05, *p*=0.116) and 0.71 (95% CI 0.55–0.93, *p*=0.013) for time to virologic failure and treatment switch. When the transition period yield was excluded, the sample size decreased to 4613 patients, and aHRs were 0.65 (95% CI 0.44–0.96, *p*=0.032) and 0.84 (95% CI 0.53–1.34, *p*=0.470). When follow-up time was restricted to two years, the aHRs were 0.77 (95% CI 0.61–0.98, *p*=0.033) and 0.69 (95% CI 0.52–0.93, *p*=0.016), respectively. When including an extra category for missing CD4 values, the sample sizes increased to 6840 and 6847, and the aHRs were 0.79 (95% CI 0.64–0.96, *p*=0.018) and 0.74 (95% CI 0.59–0.93, *p*=0.010).

## Discussion

This study investigated the effect of earlier first VL measurements and treatment outcomes. Our findings suggest that VL measurements at three months after ART initiation decrease the risk of virologic failure and treatment switching compared to first VL measurements at six months. Each month delay of the first VL led to a 9% increase in risk of virologic failure and a 14% increased risk of switching to a second-line regimen.

In this study, VL at three months was used to monitor early adherence and target adherence interventions, rather than solely to detect virologic failure. The relationship between good adherence and virologic suppression is well established [7,22–24], and a previous study from the same setting highlighted that early adherence after ART initiation determines long-term virologic suppression [10]. Second, given that early virologic breakthrough is mainly due to poor adherence and treatment interruptions rather than virologic resistance [25], adherence problems need to be detected early after treatment initiation before the emergence of resistant strains. In a study from South Africa, 53% of patients with elevation in VL at four months after ART initiation who underwent targeted adherence interventions did not progress to virologic failure: only an estimated 5.6% had confirmed virologic failure at 32 months [12].

Sub-optimal virologic suppression results in the emergence of drug-resistant HIV strains and leads to switch to second-line ART. Given that patients on second-line ART have an increased risk of treatment failure when compared to first-line ART [3], premature virologic failure and abandonment of potent first-line regimens has to be avoided where third-line therapy options are limited. Current third-line regimens containing darunavir, raltegravir or tipranavir cost approximately 1600 US dollars per patient per year in South Africa, up to 14 times the price of the current recommended first-line regimen containing tenofovir, lamivudine and EFV/NVP.

The WHO public health approach to ART delivery states that lack of laboratory testing should not be a barrier to wide-scale ART provision, but recommends that countries should begin to phase in VL capability as the preferred strategy for monitoring treatment response [26]. In case of availability of routine VL testing, the first measurement is suggested at six months [8]. In order to scale up access to VL, there is a need for less complex and less expensive VL testing, including point of care for certain settings, for a maximized public health approach.

A weakness of our study is the observational study design. However, we used DAGs, restriction and sensitivity analyses to address confounding and selection bias. DAGs, recommended for retrospective analysis of databases [27], explicitly describe the relationships between potential confounders, and help to determine *a priori* the most appropriate set of confounding variables to be included in regression analysis [28–32]. We restricted the study population to patients with an outcome or censoring after nine months of ART initiation. Restriction was applied to ensure that patients in VL3M and VL6M were comparable with regard to time at risk. Furthermore, early treatment switches are more likely related to factors such as drug toxicities than virologic failure. High proportions of early mortality and LTFU, as reported from different settings [33–35], may confound the association between the intervention and the outcome as they are competing risk factors for the outcome (survival bias). VL monitoring does not allow detecting non-adherence in real time [26]. Real-time treatment adherence data (such as pill counts, patient self-reports, pharmacy drug refill records) were not recorded consistently in our setting, and could not be utilized as covariates in the models to assess their effect on timing of VL testing. Finally, sensitivity analysis addressed the possibility of indication and patient self-selection bias. In 2005, national treatment guidelines changed to recommend the first VL at six months. The transition period was prolonged without clear cut-off date. Clinicians chose more freely the timing of the first VL, probably dependent on the perceived risk of the patient to have the outcome of interest. This potentially introduced an erroneous relationship between the intervention and outcome. In addition, patients with a VL at six months in earlier calendar periods may have missed appointments for various unknown reasons, and patients with a VL at three months in the later years might have been more motivated to know their VL.

Misclassification of the outcome variables virologic failure and switch to second-line ART is another potential weakness. For instance, Boulle *et al*. [3] reported that 26% of patients on a second-line ART regimen did not have virologic failure according to guideline definition. The definitions and tools used to assess virologic failure may miss some cases of low-level viraemia, or VLs might not have been done or captured. In addition, patients may have been switched for other reasons than failure (such as drug toxicity), and not everyone with real treatment failure was switched. Routine VL monitoring is the gold standard to assess treatment failure in a resource-constrained context, since immunological and clinical criteria poorly predict true failure [9,36–40]. Potential misclassification here would be non-differential between groups, biasing the effect measure towards the null. Hence, this study might underestimate the effect of earlier VL measurement. Finally, a single elevated VL which can be due to antiretroviral resistance or poor treatment adherence may overestimate the need for treatment switching. More than half of patients with one high VL can be virologically suppressed at retesting [41]. Therefore, we applied the WHO definition of virologic failure, requiring two consecutive elevated VLs within 3–6 months with adherence intervention between measurements [26].

Finally, temporal trends could have affected our findings, especially in view of the fact that the number of patients receiving ART in these clinics increased over time. In our study, most of the three months VLs were done in earlier calendar years. Changes in quality of care over time could have affected the findings in both directions. Virologic follow-up of patients may have deteriorated in the later study years, potentially leading to decreasing VL completion rates and detection of virologic failure over time. Thus, the benefit of the early VL in the earlier years might be underestimated due to poorer ascertainment of failure in the later years.

A key strength of the study was the sensitivity analyses, which did not change our overall conclusion. The estimated effects remained in the same range, though not all of them significant. This was probably due to decreased power and precision caused by categorization and a one third smaller sample size when excluding the transition year and VLs not done according to the guideline. Another strength was the large sample size, which allowed us to observe a large number of events. The analysis included a broad range of adult patients in different advanced stages of disease severity, and the patient sample is representative for large primary care ART clinics in urban settings. Also because findings were obtained from three large public health sectors clinics, this cohort and programme implementation are comparable to other public sector scale-up treatment programmes in Southern Africa, using routine VL monitoring to assess treatment adherence and/or virologic failure.

## Conclusions

In summary, the use of early VL monitoring at three months to identify sub-optimal adherence and to target enhanced adherence support increases medium- to long-term virologic suppression and decreases switching to second-line ART. It can contribute to the long-term success of large ART programmes in resource-constrained settings. A single early VL measurement at three months could be considered in contexts where VLs are not used regularly, yet the impact of such a strategy needs to be investigated, as it could lead to unnecessarily switches to second-line ART.

## Supplementary Material

Superior virologic and treatment outcomes when viral load is measured at 3 months compared to 6 months on antiretroviral therapy

Superior virologic and treatment outcomes when viral load is measured at 3 months compared to 6 months on antiretroviral therapy
