# Supplementary material for: Superior virologic and treatment outcomes when viral load is measured at 3 months compared to 6 months on antiretroviral therapy
Source: J Int AIDS Soc. 2015 Sep 23;18(1):20092. doi: 10.7448/IAS.18.1.20092 (PMC4582072; doi:10.7448/IAS.18.1.20092)
Supplement: Superior virologic and treatment outcomes when viral load is measured at 3 months compared to 6 months on antiretroviral therapy [file JIAS-18-20092-s001.pdf]

Additional file 1. Unadjusted and adjusted hazard ratios of virologic failure (n= 6264).

	Univariate analysis			Multivariate analysis PHA (p=0.493), VIF*** (1.01-1.16)		
	**cHR***	**95% CI**	**p**	**aHR****	**95% CI**	**p**
**VL group**						
6 months	1		0.257	1		0.016
3 months	1.10	0.93-1.29		0.78	0.64-0.95	
**Sex**						
Female	1		0.058	1		0.767
Male	0.84	0.70-1.01		0.97	0.80-1.18	
**Age**[1]	0.71	0.63-0.79	<0.001	0.72	0.64-0.81	<0.001
**CD4 count**[2]	0.90	0.85-0.95	<0.001	0.91	0.85-0.96	0.001
**WHO stage**						
I + II	1		0.001	1		0.001
III + IV	1.50	1.19-1.89		1.57	1.21-2.03	
**Clinic**						
I	1		0.28	1		0.437
II	0.96	0.78-1.19		0.92	0.73-1.14	
III	1.12	0.93-1.34		1.08	0.90-1.30	
**Baseline NNRTI**						
Nevirapine	1		0.004	1		<0.001
Efavirenz	0.79	0.68-0.93		0.73	0.61-0.87	
**ART initiation**[3]						
	0.97	0.95-0.98	<0.001	0.97	0.95-0.98	<0.001

* cHR, crude hazard ratio

** aHR, adjusted hazard ratio

*** PHA, p value for the global proportional-hazards assumption; VIF, range of variance inflation factors

[1] per 10 years

[2] per 50 cells/μl

[3] Calendar time of ART initiation; per 90 days
