# Supplementary material for: Superior virologic and treatment outcomes when viral load is measured at 3 months compared to 6 months on antiretroviral therapy
Source: J Int AIDS Soc. 2015 Sep 23;18(1):20092. doi: 10.7448/IAS.18.1.20092 (PMC4582072; doi:10.7448/IAS.18.1.20092)
Supplement: Superior virologic and treatment outcomes when viral load is measured at 3 months compared to 6 months on antiretroviral therapy [file JIAS-18-20092-s002.pdf]

Additional file 2. Unadjusted and adjusted hazard ratios of treatment switching (n= 6269).

		**Univariate analysis**		**Multivariate analysis PHA (p=0.187), VIF*** (1.01-1.23)**		
	**cHR***	**95% CI**	**p**	**aHR****	**95% CI**	**p**
**VL group**						
6 months	1		0.803	1		0.008
3 months	1.02	0.85-1.23		0.73	0.58-0.92	
**Sex**						
Female	1		0.006	1		0.150
Male	0.75	0.60-0.92		0.85	0.67-1.06	
**Age**[1]						
16-24	1		<0.001	1		<0.001
25-34	0.49	0.39-0.62		0.48	0.38-0.62	
35-44	0.38	0.29-0.50		0.38	0.28-0.51	
45-60	0.31	0.21-0.48		0.34	0.22-0.52	
**CD4 count**[2]	0.84	0.78-0.90	<0.001	0.85	0.79-0.91	<0.001
**WHO stage**						
I + II	1		0.004	1		0.001
III + IV	1.48	1.13-1.94		1.69	1.24-2.29	
**Clinic**						
I	1		<0.001	1		0.005
II	0.76	0.61-0.94		0.72	0.57-0.90	
III	0.48	0.38-0.59		0.46	0.37-0.58	
**Baseline NNRTI**						
Nevirapine	1		0.089	1		0.008

Efavirenz	0.86	0.71-1.02		0.76	0.63-0.93	
**ART initiation[3]**	0.97	0.96-0.99	0.001	0.97	0.95-0.99	0.009

* cHR, crude hazard ratio

** aHR, adjusted hazard ratio

*** PHA, p value for the global proportional-hazards assumption; VIF, range of variance inflation factors

[1] The variable age was categorized because the proportional-hazards assumption (PHA) was violated.

[2] per 50 cells/µl

[3] Calendar time of ART initiation; per 90 days
